# Evaluation of soluble transferrin receptor and malondialdehyde levels associated with ferroptosis in children with familial Mediterranean fever in remission

**DOI:** 10.1590/1806-9282.20252110

**Published:** 2026-06-15

**Authors:** Seyda Dogantan, Adem Keskin, Burcu Bozkaya Yücel, Peren Perk, Arzu Şekerci Yüksel, Rahime Koç

**Affiliations:** 1 Başakşehir Çam ve Sakura City Hospital, Department of Pediatric Rheumatology – Istanbul, Turkey.; 2 Aydın Adnan Menderes University, Faculty of Medicine, Department of Medical Biochemistry – Aydın, Turkey.; 3 Samsun Research and Education Hospital, Department of Pediatric Rheumatology – Samsun, Turkey.; 4 Başakşehir Çam ve Sakura City Hospital, Department of Pediatric Neurology – Istanbul, Türkiye.; 5 Başakşehir Çam ve Sakura City Hospital, Department of Medical Biochemistry – İstanbul, Türkiye.

**Keywords:** Familial Mediterranean fever, Ferroptosis, Lipid peroxidation, Malondialdehyde, Children

## Abstract

**OBJECTIVE:**

Familial Mediterranean fever, an autoinflammatory disease, is characterized by recurrent stereotypic febrile attacks associated with an inflammatory syndrome. Malondialdehyde, a lipid peroxidation marker, and soluble transferrin receptor, a key regulator of cellular iron homeostasis, are important markers associated with ferroptosis. The aim of this study was to investigate the levels of these markers in children with familial Mediterranean fever in remission.

**METHODS:**

The study included 30 pediatric familial Mediterranean fever patients in remission who were being monitored in the hospital and 30 healthy children. Malondialdehyde and soluble transferrin receptor levels were analyzed using enzyme-linked immunosorbent assay. Malondialdehyde and soluble transferrin receptor levels were compared between groups. Correlation analyses were also performed separately for each group to assess the relationship between these two parameters.

**RESULTS:**

Serum amyloid A, erythrocyte sedimentation rate, and creatinine levels were higher and hematocrit levels were lower in the familial Mediterranean fever group compared to the control. No significant difference was found in malondialdehyde and soluble transferrin receptor levels between the two groups. There was a positive correlation between malondialdehyde and soluble transferrin receptor levels in both groups (familial Mediterranean fever r=0.803, control r=0.599).

**CONCLUSION:**

Although there was no significant difference in mean malondialdehyde and soluble transferrin receptor levels between children with familial Mediterranean fever in remission and a healthy control group, the high correlation coefficient between these two parameters in familial Mediterranean fever patients suggests a possible link between iron imbalance and oxidative stress in the pathophysiology of the disease. These findings highlight the need for larger, multicenter studies, including patients in the exacerbation phase, to better elucidate the role of ferroptosis in familial Mediterranean fever.

## INTRODUCTION

Familial Mediterranean fever (FMF) is the most common monogenic autoinflammatory disease worldwide. It is characterized by recurrent stereotypical febrile attacks lasting less than 3 days and primarily affects children of Mediterranean origin. These attacks are usually associated with acute abdominal pain and biological inflammatory syndrome. Diagnosis is usually confirmed by mutations in the Mediterranean fever (MEFV) gene, particularly in exon 10^1^. Although FMF is considered an episodic disease characterized by short-term attacks, various chronic inflammatory conditions associated with this disease have also been described^2^. Additionally, in FMF, acute phase reactants increase during attacks as a sign of the inflammatory response, leading to increased exposure to oxidative stress^3^.

Cell death refers to the cessation of a cell's existence and is an inevitable and critical event in the life cycle, regardless of whether it occurs under normal or pathological conditions. Unlike apoptosis, autophagy, and necrosis, ferroptosis is a unique form of iron-dependent programmed cell death^4^. Cellular redox homeostasis is regulated by various metabolic pathways, including iron, glucose, lipid, and amino acid metabolisms. Key features of ferroptosis include iron-dependent lipid peroxidation, decreased glutathione peroxidase 4 activity, and reactive oxygen species (ROS)-mediated lipid peroxidation^5^. Recent studies have shown that ferroptosis and oxytosis are not entirely independent mechanisms but share some common biological processes. Lipoxygenase enzymes are involved in both cell death processes, excessive ROS production triggers the process, and similar changes in the expression patterns of some genes have been reported^6^.

Malondialdehyde (MDA) is indicative of lipid peroxidation, which is generally defined as a process in which oxidants, such as free radicals, attack lipids containing carbon–carbon double bonds, particularly polyunsaturated fatty acids^7^. Furthermore, MDA accumulation is an important biomarker that supports the induction of ferroptosis through mitochondrial structural changes, lipid-induced ROS production, and increased iron overload^8^. On the other hand, a recent study evaluating lipid metabolism in FMF patients reported that children with FMF had higher total cholesterol levels, and that high cholesterol levels were associated with an increase in symptom days^9^.

The soluble transferrin receptor (sTfR), a key regulator of cellular iron homeostasis, is the proteolytically cleaved form of the tissue receptor, the product of the human *TfR* gene. sTfR levels are used to distinguish iron deficiency from anemia of chronic disease in inflammatory conditions and to monitor the effectiveness of erythropoietin therapy. sTfR concentrations provide information about both iron metabolism and erythropoiesis by reflecting the receptor density on cells and the number of cells expressing the receptor (erythropoietic activity)^10^. However, sTfR is considered a more reliable biomarker than ferritin and transferrin saturation, which change significantly in the presence of inflammation. This is particularly important for accurate assessment in clinical situations where iron imbalance can lead to cellular damage through the production of toxic oxygen radicals and ferroptosis^11^. On the other hand, chronic and subclinical inflammation beyond acute attacks is a well-known feature of FMF; high levels of acute phase reactants, pro-inflammatory cytokines, and other inflammatory biomarkers persist even during non-attack periods^12^.

Beyond acute inflammatory attacks, FMF results from dysregulated activation of the pyrin inflammasome, leading to overproduction of pro-inflammatory cytokines such as interleukin-1β and persistent subclinical inflammation^13^. Pyrin inflammasome activation has been shown to increase the production of mitochondrial ROS, disrupting mitochondrial function and cellular redox homeostasis^14^. Mitochondria play a central role in iron processing, lipid metabolism, and oxidative balance, all of which are critical determinants of ferroptosis^15^. Furthermore, chronic inflammatory signaling can disrupt iron homeostasis by altering iron uptake and utilization pathways, thereby increasing intracellular unstable iron pools and enhancing susceptibility to lipid peroxidation^16^. In this context, persistent oxidative stress, mitochondrial dysfunction, and iron dysregulation associated with pyrin inflammasome-mediated inflammation can create a biological environment that predisposes patients.

The aim of this study was to investigate sTfR and MDA levels as biomarkers associated with ferroptosis in children with FMF. Given that FMF is characterized by recurrent inflammatory attacks accompanied by oxidative stress and that ferroptosis is a form of iron-dependent programmed cell death driven by lipid peroxidation and ROS production, assessment of sTfR and MDA may provide new insights into the possible mechanisms of iron imbalance, oxidative damage, and ferroptosis in the pathogenesis of FMF.

## METHODS

### Study design

The study was approved by the Ethics Committee of Başakşehir Çam and Sakura Hospital (Approval Number: KAEK-11/10.09.2025.333). In total, 30 FMF patients followed up in the Pediatric Rheumatology Clinic were included in the study as the FMF group. FMF diagnoses were confirmed according to the Eurofever/Pediatric Rheumatology International Research Organization (PRINTO) criteria^17^. All children diagnosed with FMF were receiving colchicine. Participants older than 18 years or younger than 2 years, those with proteinuria or amyloidosis, those with any chronic comorbidities, especially those with concurrent hematologic abnormalities, those with active infections, and those receiving corticosteroid treatment were excluded from the research. Additionally, 30 healthy children without chronic illness, malignancy, inflammatory or hematological abnormalities, or medication use were included in the study as the control group.

Sample size calculations were performed using G*Power 3.1.9.2 software. In a post-power analysis to compare two independent groups, the statistical power of the study was calculated to be approximately 61% under the assumptions of effect size d=0.50 and α=0.05. This finding indicates that the current sample size is limited in detecting moderate and especially small effect sizes, but may have better sensitivity for larger effect sizes.

### Soluble transferrin receptor and malondialdehyde analysis

The sTfR and the MDA levels were measured by enzyme-linked immunosorbent assay (ELISA). These assessments were performed using the Human Soluble Transferrin Receptor (sTfR) ELISA kit (catalog no. 201-12-0282) and the Human Malondialdehyde (MDA) ELISA kit (catalog no. 201-12-1372), both supplied by SunRed Biological Technology Co., Ltd (Shanghai, China).

### Statistical analysis

For statistical analyses, IBM SPSS Statistics 22 (IBM Corp., Armonk, NY, USA) software was used for Windows. The conformity of continuous variables to normal distribution was assessed using the Shapiro-Wilk test, along with skewness and kurtosis values. Homogeneity of variance was tested using the Levene's test. Continuous variables are presented as mean±standard deviation (X±SD). The independent sample t-test was used to compare continuous variables with normal distribution, and the Mann-Whitney U test was used for variables without normal distribution. Spearman correlation analysis was used to evaluate the relationship between variables. Statistical significance was accepted as p<0.05 in all analyses.

## RESULTS

In total, 30 FMF patients aged 2–17 were included in the study as the FMF group. Also, 30 healthy children aged 6–17 were included in the control group. The mean age of the FMF group was 10.13±3.99 years, and the mean age of the control group was 10.66±3.21 years. The proportion of female children in the FMF group was 43.33% (n=13), and the proportion of female children in the control group was 66.67% (n=20). No significant difference was found between the two groups in terms of mean age and gender ratios (p=0.570 and p=0.069, respectively). Descriptive information for the FMF group is presented in Table 1.

**Table 1:** Demographic and clinical information of the familial Mediterranean fever group.

		FMF group (n=30)
Height (cm) X±SD		138.17±20.55
Weight (Kg) X±SD		34.50±15.58
Body mass index (kg/cm^2^) X±SD		17.09±3.38
Disease age (ay) X±SD		43.29±51.27
Family history n (%)		22 (73.33)
Consanguineous marriage n (%)		9 (30)
Rheumatic disease in family n (%)		7 (23.33)
Symptom duration in days	1	4 (13.33)
	2	9 (30)
	3	13 (43.33)
	4	3 (10)
	7	1 (03.33)
Average of day of symptoms X±SD		2.67±1.18

FMF: familial Mediterranean fever; SD: standard deviation.

A family history of FMF was found in 73.33% (n=22) of the patients, and consanguineous marriage was found in 30% (Table 1). Furthermore, the most common symptom duration was 3 days (43.33%) (Table 1).

Laboratory results for the FMF and control groups are presented in Table 2. Mean serum amyloid A, ESR, and creatinine levels were higher in the FMF group than in the control group, while hematocrit levels were lower in the FMF group (Table 2). No significant difference was found between the two groups in terms of malondialdehyde and sTfR levels (Table 2).

**Table 2:** Laboratory findings of the groups.

Laboratory findings	FMF group (n=30)	Control group (n=30)	p
MDA (nmol/mL)	24.4±24.19	17.86±6.85	0.164
sTfR (mg/L)	1.02±0.79	0.77±0.3	0.117
Serum amyloid A (mg/L)	8.17±17.71	0.88±0.66	0.032
ESR (mm/hour)	12.07±9.78	2.87±1.81	<0.001
CRP (mg/L)	13.2±31.53	1.76±1.81	0.052
WBC (x10^3^/μL)	7.33±1.7	7.61±1.81	0.532
Hematocrit (%)	36.82±3.04	40.86±10.55	0.049
Hemoglobin (g/dL)	12.31±1.16	12.21±2.43	0.837
Neutrophile (x10^3^/μL)	3.8±1.63	3.77±1.32	0.936
Lymphocyte (x10^3^/μL)	2.74±0.77	2.98±0.74	0.232
Platelet (x10^3^/μL)	319.27±90.98	336.03±98.4	0.496
MCV (fL)	77.99±3.83	79.03±4.16	0.319
BUN (mg/dL)	22.78±5.71	24.88±4.92	0.132
Creatinine (mg/dL)	0.54±0.15	0.33±0.13	<0.001
ALT (U/L)	14.8±5.12	15.7±7.4	0.586
AST (U/L)	23.47±6.1	23±7.58	0.794
LDH (U/L)	223.9±49.5	219.53±48.46	0.731
Vitamin B12 (pg/mL)	370.93±298.67	–	–
Vitamin D (ng/mL)	21±8.44	–	–
Folic acid (ng/mL)	7.53±3.79	–	–
Total protein (g/L)	72.8±4.41	–	–
Albumin (g/L)	45.83±3.53	–	–
T4 (ng/dL)	1.4±0.23	–	–
TSH (mIU/L)	2.31±1.09	–	–
Calcium (mg/dL)	9.73±0.44	–	–
Magnesium (mg/dL)	2.06±0.11	–	–

MDA: malondialdehyde; sTfR: soluble transferrin receptor; ESR: erythrocyte sedimentation rate; WBC: white blood cell; CRP: C-reactive protein; MCV: mean corpuscular volume; BUN: blood urea nitrogen; ALT: alanine transaminase; AST: aspartate aminotransferase; LDH: lactate dehydrogenase; TSH: thyroid-stimulating hormone; FMF: familial Mediterranean fever.

A positive correlation was found between malondialdehyde and sTfR levels in both groups (FMF group r=0.803, p<0.001, control group r=0.599, p<0.001).

In the FMF group, correlation analysis performed to evaluate the relationship between malondialdehyde and sTfR levels and symptom duration (in days) revealed no statistically significant association (malondialdehyde r=0.278, p=0.136, sTfR r=0.140, p=0.461). Additionally, in the FMF group, malondialdehyde and sTfR levels were evaluated according to the patients’ descriptive characteristics, and the results are presented in Table 3.

**Table 3:** Malondialdehyde and soluble transferrin receptor levels according to the descriptive characteristics of the familial Mediterranean fever group.

Patients’ descriptive characteristics		MDA (nmol/mL)	sTfR (mg/L)
Family history	Yes (n=22)	17.57±8.83	0.79±0.38
	No (n=8)	43.18±40.51	1.65±1.24
	p	0.133	0.067
Consanguineous marriage	Yes (n=9)	33.77±28.80	1.39±0.97
	No (n=21)	20.38±21.45	0.86±0.67
	p	0.118	0.213
Rheumatic disease in family	Yes (n=7)	46.46±42.35	1.71±1.33
	No (n=23)	17.68±8.96	0.81±0.38
	p	0.095	0.148

MDA: malondialdehyde; sTfR: soluble transferrin receptor.

## DISCUSSION

Accumulating evidence suggests that oxidative stress may play an important role in the pathophysiology of FMF, particularly in relation to recurrent inflammatory attacks. During these attacks, oxidative stress increases, and this increase is associated with an acute phase response and increased free radical production. Increased free radicals can lead to lipid peroxidation and iron imbalance at the cellular level^3,18-20^. Ediz et al. reported that MDA levels increased during attacks in adult FMF patients, while antioxidant parameters remained unchanged^18^. Yucel and colleagues reported higher oxidative stress parameters in pregnant women with FMF^19^. In contrast, no difference in MDA levels was found between adult FMF patients in remission and healthy individuals^21^.

In this study, no difference in MDA levels was found between children in FMF remission and healthy children. This situation may be due to the fact that children with FMF are receiving colchicine treatment. Indeed, colchicine has known anti-inflammatory and potentially antioxidant properties. In addition, the fact that the patients are in remission may have masked pathophysiological processes such as acute oxidative stress and irregularities in iron metabolism. On the other hand, according to the Shapiro-Wilk test results, it was observed that MDA levels in children with FMF deviated statistically significantly from the normal distribution compared to healthy children.

Sahin et al. reported that colchicine has a protective effect against oxidative stress by regulating the release of vitamin E, β-carotene, and calcium in FMF patients in remission^22^. A recent study found that patients with vitamin D deficiency had higher total oxidant levels. However, there was no significant difference between patients with vitamin D insufficiency and those with optimal vitamin D levels^23^. On the other hand, a recent study with a very large sample size defined vitamin D levels below 20 ng/mL as vitamin D deficiency, 20–29 ng/mL as vitamin D insufficiency, and 30–100 ng/mL as optimal vitamin D levels^24^. A recent study reported that omega-3 (anti-inflammatory) and Alaskan blueberry extract (antioxidant) supplements given to FMF patients were associated with a parallel significant reduction in MDA levels and inflammatory markers, improved endothelial function, and improved oxidative status after 24 weeks of treatment^20^.

In this study, which included children with FMF who were followed up, all patients were receiving colchicine treatment. Furthermore, the mean vitamin D levels in these patients were found to be in the vitamin D insufficiency range. The reason that MDA levels were not statistically higher in children with FMF compared to healthy children may also be related to vitamin D levels at the time of colchicine treatment.

Cell death programs such as ferroptosis and apoptosis are linked to abnormal redox homeostasis associated with lipid metabolism and membrane functions, which increases the susceptibility of cells to oxidative membrane damage^25^. In a study involving 172 pediatric patients diagnosed with FMF, it was reported that there was no significant difference in sTfR levels, a marker of cellular iron requirement, between newly diagnosed patients and patients receiving colchicine treatment. Similarly, no differences were reported in interleukin-6 and iron parameters^26^. On the other hand, in FMF, which is characterized by inflammatory attacks, interleukin-6 stimulation causes an increase in hepcidin levels, a protein associated with iron metabolism. However, a study by Asan and colleagues found that serum hepcidin levels in FMF patients were lower than in healthy individuals. The researchers explained this finding by noting that only FMF patients who were not in the attack phase were included in the study^27^.

In this study, no significant difference was found in sTfR levels between children with FMF and healthy children. This may be because the patients were evaluated during remission. On the other hand, a relatively high positive correlation has been found between sTfR and MDA levels in children with FMF, even when they are in remission.

In this study, SAA, ESR, and creatinine levels were found to be significantly higher in patients with FMF compared to healthy children. However, no significant difference was found between the groups in terms of CRP levels. This may be related to the non-normal distribution of CRP levels in children with FMF, which exhibits a highly heterogeneous distribution. It may also be because the patients were evaluated during the remission period.

This study has some limitations. First, the limited sample size is one of the most significant limitations of the study. However, it is thought that the findings will serve as a reference for future studies with larger sample sizes. Another limitation of the study is that it was conducted only with patients in remission. However, the fact that the findings will serve as a comparative reference for future studies that also include individuals in the active phase of the disease is considered one of the strengths of the study. Finally, the lack of biomarkers related to iron metabolism is a significant limitation that restricts the biological interpretation of sTfR results.

## CONCLUSION

No significant difference was observed in mean sTfR and MDA levels between FMF patients in remission and a healthy control group. This may be related to the patients being in remission and receiving colchicine treatment. On the other hand, the results suggest that evaluation with larger sample groups is needed in the active phase of FMF in children. Additionally, a positive correlation was observed between these two parameters in children with FMF; however, this finding alone is not sufficient to establish a definitive pathophysiological link. Further studies at the molecular level are needed to elucidate the potential biological mechanisms supporting this relationship.

## Data Availability

The datasets generated and/or analyzed during the current study are available from the corresponding author upon reasonable request.
